# Prediction of lncRNA–Disease Associations *via* Closest Node Weight Graphs of the Spatial Neighborhood Based on the Edge Attention Graph Convolutional Network

**DOI:** 10.3389/fgene.2021.808962

**Published:** 2022-01-04

**Authors:** Jianwei Li, Mengfan Kong, Duanyang Wang, Zhenwu Yang, Xiaoke Hao

**Affiliations:** ^1^ Institute of Computational Medicine, School of Artificial Intelligence, Hebei University of Technology, Tianjin, China; ^2^ Hebei Province Key Laboratory of Big Data Calculation, Hebei University of Technology, Tianjin, China

**Keywords:** lncRNA–disease association prediction, graph convolutional network, heterogeneous networks, graph of the spatial neighborhood, correlation score

## Abstract

Accumulated evidence of biological clinical trials has shown that long non-coding RNAs (lncRNAs) are closely related to the occurrence and development of various complex human diseases. Research works on lncRNA–disease relations will benefit to further understand the pathogenesis of human complex diseases at the molecular level, but only a small proportion of lncRNA–disease associations has been confirmed. Considering the high cost of biological experiments, exploring potential lncRNA–disease associations with computational approaches has become very urgent. In this study, a model based on closest node weight graph of the spatial neighborhood (CNWGSN) and edge attention graph convolutional network (EAGCN), LDA-EAGCN, was developed to uncover potential lncRNA–disease associations by integrating disease semantic similarity, lncRNA functional similarity, and known lncRNA–disease associations. Inspired by the great success of the EAGCN method on the chemical molecule property recognition problem, the prediction of lncRNA–disease associations could be regarded as a component recognition problem of lncRNA–disease characteristic graphs. The CNWGSN features of lncRNA–disease associations combined with known lncRNA–disease associations were introduced to train EAGCN, and correlation scores of input data were predicted with EAGCN for judging whether the input lncRNAs would be associated with the input diseases. LDA-EAGCN achieved a reliable AUC value of 0.9853 in the ten-fold cross-over experiments, which was the highest among five state-of-the-art models. Furthermore, case studies of renal cancer, laryngeal carcinoma, and liver cancer were implemented, and most of the top-ranking lncRNA–disease associations have been proven by recently published experimental literature works. It can be seen that LDA-EAGCN is an effective model for predicting potential lncRNA–disease associations. Its source code and experimental data are available at https://github.com/HGDKMF/LDA-EAGCN.

## Introduction

Long non-coding RNAs (lncRNAs) are a large and important class of non-coding RNAs with a molecular length more than 20 nucleotides (Ponting et al., 2009). In recent years, more and more biological experiments and clinical studies have demonstrated that lncRNAs participate in almost all the stages of organism life, from regulating single cell life span to maintaining the homeostasis stability of the whole organism, which are closely implicated in the occurrence and development of various complex human diseases. Many human diseases are caused by the dysfunctions of lncRNAs or their abnormal expressions that are reflected in the associations between lncRNAs and diseases (Kapranov et al., 2007; Mercer et al., 2009; Guttman et al., 2013). Therefore, the studies of lncRNA–disease associations are helpful to deeply understand the pathogenesis of complex human diseases at the molecular level and would be increasingly used to aid in the prevention, diagnosis, and treatment of diseases (Wang and Chang, 2011). Due to the high cost of traditional biological experiments of identifications of lncRNAs, there are only a relatively limited number of known lncRNA–disease associations that have been confirmed; thus, identifying potential lncRNA–disease associations has become a hot topic through computational models in the fields of human complex diseases.

Nowadays, many computational models based on integrating a vast amount of heterogeneous biological data have been proposed to predict novel lncRNA–disease associations. Broadly, they can be categorized into two types. The models in the first category are based on homogeneous or heterogeneous biological information networks. For example, Liao et al. (2011) constructed a coding–non-coding gene co-expression network for predicting probable functions for altogether 340 lncRNAs based on topological or other network characteristics. Yang et al. (2014) developed a coding–non-coding gene–disease bipartite network based on the known associations between diseases and disease-causing genes, and applied a propagation algorithm mining 768 potential lncRNA–disease associations in the constructed network. Sun et al. (2014) proposed a global network–based model, RWRlncD, which inferred lncRNA–disease associations with the random walk with a restart algorithm of the lncRNA functional similarity network. However, RWRlncD cannot be applied to the diseases which have no verified association with any lncRNA. Chen et al. (2016) reported an improved random walk with the restart model, IRWRLDA, which could be applied to diseases without any known related lncRNAs through setting the initial probability vector. Fu et al. (2018) predicted lncRNA–disease associations by translating row data matrices into low-rank matrices in the heterogeneous data with matrix tri-factorization for gaining their intrinsic and shared structure. Ding et al. (2018) integrated lncRNA–disease–gene information and lncRNA–disease associations to describe the heterogeneity of coding–non-coding gene–disease association, and proposed an lncRNA–disease–gene tripartite graph to predict potential lncRNA–disease associations. Wang et al. (2019) proposed a new prediction model based on the internal inclined random walk with the restart algorithm. A novel method called network consistency projection was proposed by Xie et al. (2019), based on integrating a known lncRNA–disease association network, a lncRNA–disease cosine similarity network, and a lncRNA expression similarity network, exhibiting good predictive performance. Xie et al. (2020) developed a new method based on linear neighborhood similarity and unbalanced bi-random walk for lncRNA–disease association prediction. After the preprocessing of the lncRNA–disease association sparse matrix, an lncRNA–disease network was reconstructed according to linear neighborhood similarities. Then the unbalanced double random walk algorithm was used to calculate the prediction score. However, it is still challenging to predict potential lncRNA–disease associations accurately in the absence of the known lncRNA–disease association information.

Another major type of computational models is based on the machine learning algorithm, and the main characteristic of them is to train a classifier based on machine learning algorithms according to the biological features of lncRNAs and diseases. Chen and Yan (2013) reported a computational method of Laplacian regularized least squares for predicting lncRNA–disease associations (LRLSLDA) in a semi-supervised learning framework. In 2015, a naive Bayesian classifier–based model was proposed by Zhao et al. (2015) to predict potential lncRNA–disease associations. Chen et al. (2015) proposed two novel lncRNA functional similarity calculation models (LNCSIM), which were evaluated by introducing similarity scores into the LRLSLDA model. Lan et al. (2017) integrated a variety of gene data and trained a classifier with the bagged support vector machine for their lncRNA–disease association prediction model. Lu et al. (2018) developed a model called SIMCLDA to predict the potential lncRNA–disease associations based on the inductive complement matrix. Guo et al. (2019) proposed a LDASR model based on collaborative filtering and machine learning. Xuan et al. (2019) developed a dual convolutional neural network with attention mechanisms for predicting disease-related lncRNAs. Zeng et al. (2020) designed a hybrid computing framework called SDLDA based on linear and non-linear features of lncRNAs and diseases, and created fused features for the full connection layer for prediction. Sheng et al. (2021) constructed a deep learning prediction model, VADLP, which applied autoencoders for representation learning of lncRNA and disease features. Wu et al. (2020) adopted graph autoencoder to predict lncRNA–disease associations on lncRNA–disease bipartite graph. One of the main limits of these models based on machine learning methods is lacking the negative samples during the classifier training. For giving readers a clear overview, Supplementary File S1 induces the aforementioned models in a tabular form.

Inspired by the great success of the EAGCN method on the chemical molecule property recognition problem, the prediction of lncRNA–disease associations could be regarded as a component recognition problem in the lncRNA–disease characteristic graph. In order to fully mine core features of lncRNA–disease associations in a graph with minimum redundant features, the structure hidden in the closest node weight graph among the spatial neighborhoods of lncRNA–disease associations (CNWGSN) has been developed in this study that combined with the biological features of lncRNAs and diseases. It considered not only the features of disease–disease, lncRNA–lncRNA, and lncRNA–disease relations but also the lncRNA–disease features in a multidimensional space. Moreover, CNWGSN was used to provide a great logic and mathematical supports for the edge attention graph convolutional networks (EAGCNs) (Shang et al., 2018) for summarizing and extracting the internal features between lncRNAs and diseases. Thus, an lncRNA–disease association prediction model based on the edge attention graph convolutional network (LDA-EAGCN) was proposed; the multiple edge relations in multiple graphs of lncRNAs and diseases were used to train EAGCN in LDA-EAGCN. Additionally, to unravel the lack of negative samples for training the classifier, the network-based random walk with a restart algorithm was adopted in our study. The low score samples from lncRNA–disease associations were selected randomly as negative samples. The 10-fold cross-validations and numerical experiments illustrate that LDA-EAGCN outperformed the tested five state-of-the-art models, and the AUC value of LDA-EAGCN reached 0.9853. Moreover, the case studies of renal cell carcinoma, laryngeal cancer, and liver cancer indicated that LDA-EAGCN is capable of detecting potential lncRNA–disease associations; most of the top ten predicted lncRNAs of each case study (24 of the 30) which are most likely to have associations with the diseases have been proved by recently published experimental literature works.

## Materials and Methods

### LncRNA–Disease Associations

One dataset that is used in the study is downloaded from the Lnc2Cancer 3.0 database (Ning et al., 2016); it contains 3919 lncRNA–disease associations involving 198 diseases and 639 lncRNAs. The other dataset is downloaded from the LncRNADisease v2.0 database (Chen et al., 2013); it includes 2453 lncRNA–disease associations among 378 diseases and 472 lncRNAs. All these associations have been verified by biological experiments. In addition, a controlled and hierarchical medical vocabulary is collected from the MeSH vocabulary database (Nelson et al., 2001) for standardizing these disease names. MeSH is a biomedical subject vocabulary which has high authority in the field of medicine. After standardizing all the datasets and removing duplicated data, finally, 4715 lncRNA–disease associations of 786 lncRNAs and 292 diseases were obtained.

### LncRNA–Disease Correlation Matrix

The numbers of obtained lncRNAs and diseases are labeled as nl and nd , respectively; then the lncRNA–disease correlation matrix (LDCM) is constructed, $\mathrm{LDCM}\in R^{nl*nd}$ . The following formula can be used to calculate the value of LDCM(i,j) :

$$\text{LDCM}\left(\text{i},\text{j}\right)=\{\begin{array}{c} 1,\;\;\;\;\;\;\;\;\;\;\;\;l_{i}\;associated\;with\;d_{j}\\ 0,\;\;\;\;\;\;\;\;\;\;\;\;\;\;\;\;\;\;\;\;\;\;otherwise \end{array}.$$ (1)

In this way, the abstract correlations between lncRNAs and diseases are represented by a two-dimensional matrix which is intuitive, concise, and convenient for subsequent calculations.

### Disease Semantic Correlation

In the calculation of the semantic similarity of disease, each disease name has been represented by the MESH descriptor, and a directed acyclic graph (DAG) is structured. In the DAG, all nodes are connected by a direct edge from a more general term to a more specific term. A semantic similarity algorithm was proposed based on the hierarchical structure of disease terms (Wang et al., 2010). It makes full use of the internal branch structure of diseases, and the calculated disease similarity has sufficient theoretical support. The semantic similarity algorithm consists of three main processing steps.

Step 1: The relationship between the disease node d and the diseases in the branches involving disease d is extracted, which is named as DAG (d) . Using the extracted DAG (d) graph, the semantic contribution value $D_{d}$ is calculated according to the disease branch structure shown in DAG (d) . The shortest path from $T_{d}$ (the set of all ancestor nodes of d including d itself) to disease d in DAG (d) usually contains less branches and possesses less disease nodes in the path, which means a stronger correlation between $T_{d}$ and disease d . The semantic contribution value will be reduced at each intermediate node passing through disease d , which has been repeatedly verified by previous studies. The semantic value $D_{d}\left(t\right)$ of disease d can be calculated based on the DAG (d) :

$$\{\begin{array}{c} D_{d}\left(d\right)=1\;\;\;\;\;\;\;\;\;\;\;\;\;\;\;\;\;\;\;\;\;\;\;\;\;\;\;\;\;\;\;\;\;\;\;\;\;\;\;\;\;\;\;\;\;\\ D_{d}\left(t\right)=\max\left(\Delta *D_{d}\left(t^{\prime }\right)\text{|}t^{\prime }\in children\;of\;t\right)\;\;\;\;\;\;\;\;if\;t\neq d \end{array}.$$ (2)

The semantic contribution factor for edges linking disease t with its child disease $t^{\prime }$ is defined as Δ, which is set to 0.5 in our studies. In the DAG (d) , when there are multiple paths between $T_{d}$ and disease d, the shortest path contribution value is treated as the maximum semantic contribution value.

Step 2: Based on Eq. 2, the semantic value of disease d was calculated as Eq. 3:

$$DV\left(A\right)=\underset{t\in T_{d}}{\sum }D_{d}\left(t\right).$$ (3)

Step 3: According to the semantic values of diseases A and B , the semantic similarity value DS(A,B) of diseases A and B is calculated as Eq. 4. The common diseases of DAG (a) and DAG (b) are screened out, and their semantic contributions to diseases A and B are summed. The proportion of the semantic contribution value of the sum to the semantic value of diseases A and B is regarded as the similarity value of diseases A and B .

$$DS\left(A,B\right)=\frac{\sum_{t\in T_{A}\cap T_{B}}\left(D_{A}\left(t\right)+D_{B}\left(t\right)\right)}{DV\left(A\right)+DV\left(B\right)}.$$ (4)

Ultimately, the semantic similarity matrix of diseases is gained, and it is quick to obtain the semantic similarity between arbitrary two diseases.

### LncRNA Function Correlation

Based on the assumption that lncRNAs with similar functions may have a good likelihood of associating with similar diseases, the functional similarities of the lncRNAs can be calculated by the similarities of the diseases associated with them. Chen et al. developed novel lncRNA functional similarity calculation models for lncRNA–disease association prediction (Chen et al., 2015). In the study, these calculation models were also borrowed. $L_{m}$ denotes lncRNA *lm*, $L_{n}$ denotes lncRNA *ln*, and the diseases associated with $L_{m}$ are represented by $dm_{i}$ . All the diseases associated with $L_{m}$ become a set $DT_{m}=\left\{dm_{1},dm_{2}\ldots ,dm_{m}\right\}$ , and the diseases related to $L_{n}$ are represented by the set $DT_{n}=\left\{dn_{1},dn_{2}\ldots ,dn_{n}\right\}$ . The core idea here is to calculate the functional similarity between $L_{m}$ and $L_{n}$ by using the similarity values of diseases in $DT_{m}$ and $DT_{n}$ . First, the similarity values of disease $dm_{i}$ in $DT_{m}$ and all diseases in $DT_{n}$ are calculated in turn, and the maximum similarity value is considered as the minimum distance of the disease set $DT_{n}$ associated with disease $dm_{l}$ and ${\text{L}}_{\text{m}}$ . Second, the calculation formula for the maximum disease score $S\left(dm_{l},DT_{n}\right)$ is shown in formula Eq. 5. Similarly, the minimum distance between all diseases in the disease set $DT_{n}$ associated with $L_{n}$ and the disease set $DT_{m}$ associated with $L_{m}$ is obtained. Finally, the ratio of the maximum disease score of $DT_{m}$ and all diseases in $DT_{n}$ to the number of elements in $DT_{m}$ and $DT_{n}\;$ , respectively, is calculated, and the functional similarity score of $L_{m}$ and $L_{n}$ , $SCORE\left(L_{m},L_{n}\right)$ , is shown in formula Eq. 6:

$$S\left(dm_{l},DT_{n}\right)=\overset{1\leq i\leq n}{MAX}\left(S\left(dm_{l},dn_{i}\right)\right),$$ (5)

$$SCORE\left({\text{L}}_{m},{\text{L}}_{n}\right)=\frac{\sum_{1\leq i\leq m}S\left(dm_{i},DT_{n}\right)+\sum_{1\leq j\leq n}S\left(dt_{i},DT_{m}\right)}{m+n}.$$ (6)

The specific values of disease semantic similarity matrices and lncRNA similarity matrices are offered in Supplementary Files S2, S3, respectively.

### Negative Samples

In order to better train the LDA-EAGCN model, the random walk with restart (RWRH) algorithm was used to generate negative samples for training the prediction model based on heterogeneous networks in the study by Li and Patra (2010). This model sorts the possibilities of all associations according to the network structures and screens lncRNA–disease pairs with low correlation scores as negative samples.

The RWRH algorithm mainly consists of three steps. First, the method begins by generating the lncRNA nodes and disease nodes, and the heterogeneous network of their associations or similarities. Second, a seed node is selected as the starting node of the ergodic. Third, it is to construct the transition matrix to bridge every jump of the ergodic. Finally, negative samples in proportion to positive samples are randomly generated from lncRNA–disease pairs with low association probabilities; the detailed prediction results are provided in Supplementary File S4.

### Edge Attention Graph Convolution Networks

A convolutional neural network (CNN) is a kind of deep neural network which is widely used in biomedical relation detection. A graphical convolution neural network (GCN) is generalization of CNN to work with arbitrarily structured graphs. The edge attention–based multi-relational graph convolutional network (EAGCN) (Shang et al., 2018) is a novel model which accurately excavates multiple edge relations and extracts node features in multiple graphs.

**FIGURE 1:**
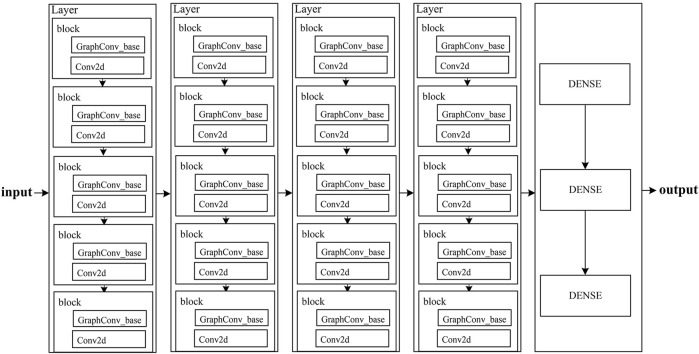
Flowchart of EAGCN method.

The flowchart of EAGCN is shown in Figure 1. It consists of four layers and three fully linked layers; each layer contains five blocks, and there are Conv2d convolution and GraphCov_base convolution based on graph convolution in each block. It was applied originally to deep learning in the chemical direction researches and directly learned the molecular properties of compounds from the molecular graphs.

In our study, the prediction of lncRNA–disease associations was treated as a binary classification problem of the component recognition based on the lncRNA–disease characteristic graph. The structural information of lncRNA–disease associations is substituted into a convolutional neural network for training the classifier of our predicting model.

### LDA-EAGCN Model

Although high-dimensional features of lncRNA–disease association have not been clearly captured and cannot be directly detected by the extractions of multilayered deep learning methods, the internal logic and rules of high-dimensional features of lncRNA–disease association would be used to predict the unknown relationships between lncRNAs and diseases. In order to introduce the EAGCN algorithm into LncRNA–disease association prediction, the graphs of lncRNA–disease association pairs were first constructed. For fully excavating internal logic features and decreasing functional redundancy of lncRNA–disease association, the structure of the closest node weight graph of the spatial neighborhood of lncRNA–disease (CNWGSN) was subsequently proposed. It combined with the biological features of lncRNAs and diseases, and can provide great logic and mathematical support for EAGCN to learn and summarize the internal relationship between lncRNAs and diseases. CNWGSN takes into account not only the features of disease–disease relationship, lncRNA–lncRNA relationship, and known lncRNA–disease associations between diseases and lncRNAs but also the known features of lncRNAs and diseases in a multidimensional feature space.

Based on the above, a novel model, LDA-EAGCN, which comprises the following three main steps was proposed.

Step 1: Structure the adjacency matrix of lncRNA–disease associations and calculate the diseases–diseases semantic correlation matrix $DDCM\in R^{dn*dn}$ and the lncRNA–lncRNA functional correlation matrix $\text{LLCM}\in R^{ln*ln}$ .

Step 2: Structure the closest node weight graph of the spatial neighborhood of lncRNA–disease (CNWGSN) of lncRNA–disease associations. It contains two classes of nodes, lncRNA l_i_ and disease d_i_, which are from the lncRNA–disease correlations (LDC). M top-ranking disease nodes, $D_{i}=\left\{d_{i1},\ldots ,d_{ii}\ldots ,d_{iM}\right\}$ , are most closely related with the disease semantics of $D_{i}$ in the disease–disease semantic correlation matrix (DDSCM), and N top-ranking lncRNA nodes, $L_{i}=\left\{l_{i1},\ldots ,l_{ii}\ldots ,l_{iN}\right\}$ , are also most closely related with the function similarities of lncRNA  i  in LLCM. In the topological sense, the closest lncRNA node weight graph (CLNWG) of lncRNA  i is constructed according to the LLCM. The M top-ranking lncRNA nodes closely related to lncRNA i are screened out to establish nodes. The weights of CLNWG are taken as the correlation values of the LLCM. In the same way, the N top-ranking disease nodes closely related to disease i are screened out to establish nodes. The weights of disease i in CLNWG are adopted as the correlation value of the DDSCM. Then the closest node weight graph of $L_{i}$ and $D_{i}$ and the spatial neighborhood features are integrated into the CNWGSN features of lncRNA  i and disease i .

The edges of CNWGSN features graph are divided into four categories. The predicted edges which need to be predicted between the input lncRNAs and the disease $\text{s}\;Core\_att\in R^{\left(N+M\right)*\left(N+M\right)*2}$ , the spatial neighborhood edges that are the association are known between diseases and lncRNAs $\;Nomal\_att\in R^{\left(N+M\right)*\left(N+M\right)*2}$ , the lncRNA edges that carry lncRNA function correlation $dd\_att\in R^{\left(N+M\right)*\left(N+M\right)*2}$ , and the disease edges that have disease semantic correlation $ll\_att\in R^{\left(N+M\right)*\left(N+M\right)*2}$ . The calculating formulas of four kinds of edges are shown as follows.

$$Core_{att\left(i,j\right)}=\{\begin{array}{c} \left(1,1\right)\;\;\;\;\;\;\;\;\;\left(i=0\;\cap j=N\right)\;\cup \;\left(i=M\;\cap \;j=0\right)\\ \left(0,0\right)\;\;\;\;\;\;\;\;\;\;\;\;\;\;\;\;\;\;\;\;\;otherwise\;\;\;\;\;\;\;\;\;\;\;\; \end{array},$$ (7)

$$Nomal\_att\left(i,j\right)=\{\begin{array}{c} \left(1,1\right)\;\;\left(d_{i\left(i-N\right)}\;associate\;to\;l_{j}\right)\cup \left(l_{i\left(i-M\right)}\;associate\;to\;d_{j}\right)\\ \left(0,0\right)\;\;\;\;\;\;\;\;\;\;\;\;\;\;\;\;\;\;\;\;otherwise \end{array},$$ (8)

$$dd\_att\left(i,j\right)=\{\begin{array}{c} \left(1,S\left(d_{i-N},d_{j-N}\right)\right)\;\;\;\;\;\;\;\;\;\;i>N\cup j>N\\ \left(0,0\right)\;\;\;\;\;\;\;\;\;\;\;\;\;\;\;\;\;\;\;\;\;\;\;\;otherwise \end{array},$$ (9)

$$ll\_att\left(i,j\right)\;=\{\begin{array}{c} \left(1,SCORE\left(l_{i},l_{j}\right)\right)\;\;\;\;\;\;\;\;\;\;\;\;\;\;i<N\cup j<N\\ \left(0,0\right)\;\;\;\;\;\;\;\;\;\;\;\;\;\;\;\;\;\;\;\;\;\;\;\;\;\;otherwise \end{array}.$$ (10)

**FIGURE 2:**
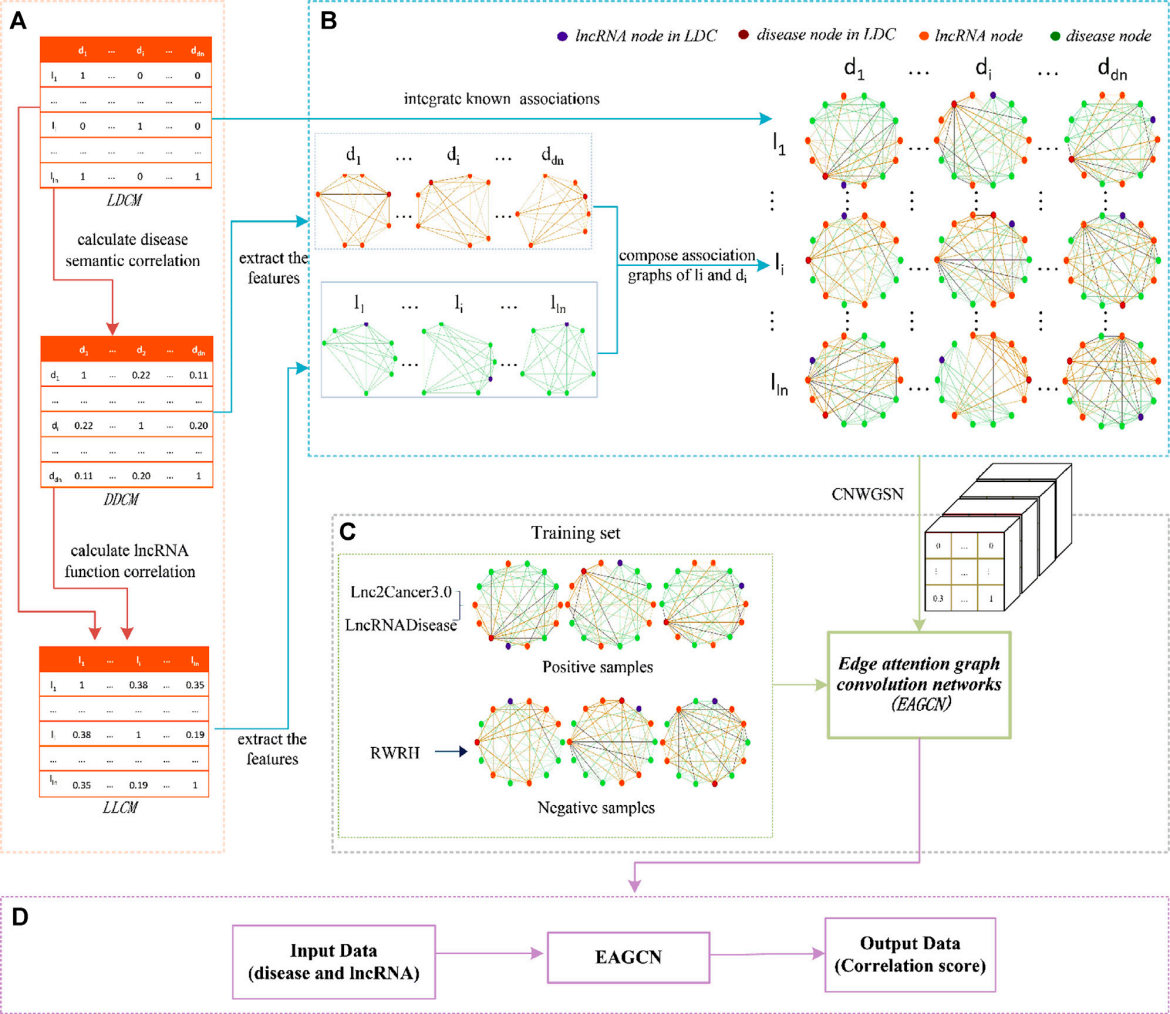
Flowchart of LDA-EAGCN. **(A)** Construction and calculation of lncRNA–disease correlation matrix (LDCM), disease–disease semantic correlation matrix (DDSCM), and lncRNA–lncRNA function correlation matrix; **(B)** constructing the closest lncRNA node weight graph (CLNWG) of the lncRNA and disease in lncRNA–disease correlations (LDCs); **(C)** training edge attention graph convolution networks (EAGCN); **(D)** predicting correlation scores of input data with EAGCN.

Step 3: The features are extracted from lncRNA–disease associations with CNWGSN, and they are treated as the training samples of EAGCN. In parallel, the constructing negative samples of lncRNA–disease associations are introduced into the training, which helps to improve the prediction accuracy of correlation scores. The flowchart of LDA-EAGCN is shown in Figure 2.

## Results

### Implementation Details of LDA-EAGCN

After specification naming and redundancy removal, all 4715 known lncRNA–disease associations were labeled as positive samples, and an equal number of negative samples with the RWRH method was constructed. These samples are included as the data of prediction performance self-assessment of the LDA-EAGCN model. During the training, the optimized parameters of the EAGCN model are adopted for avoiding the problems of overfitting and poor generalization ability, such as the packet loss rate dr = 0.3 and the learning rate α = 0.01 (for more details, see Supplementary File S5).

### Evaluation Methods and Metrics

**FIGURE 3:**
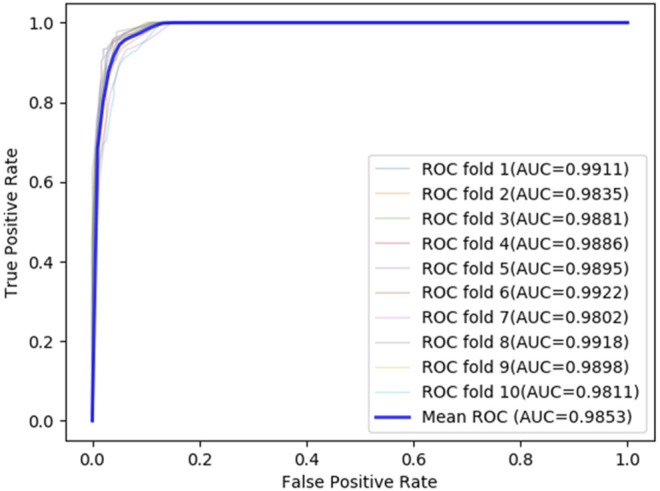
ROC curves of LDA-EAGCN in different situations of 10-fold cross-validations.

**FIGURE 4:**
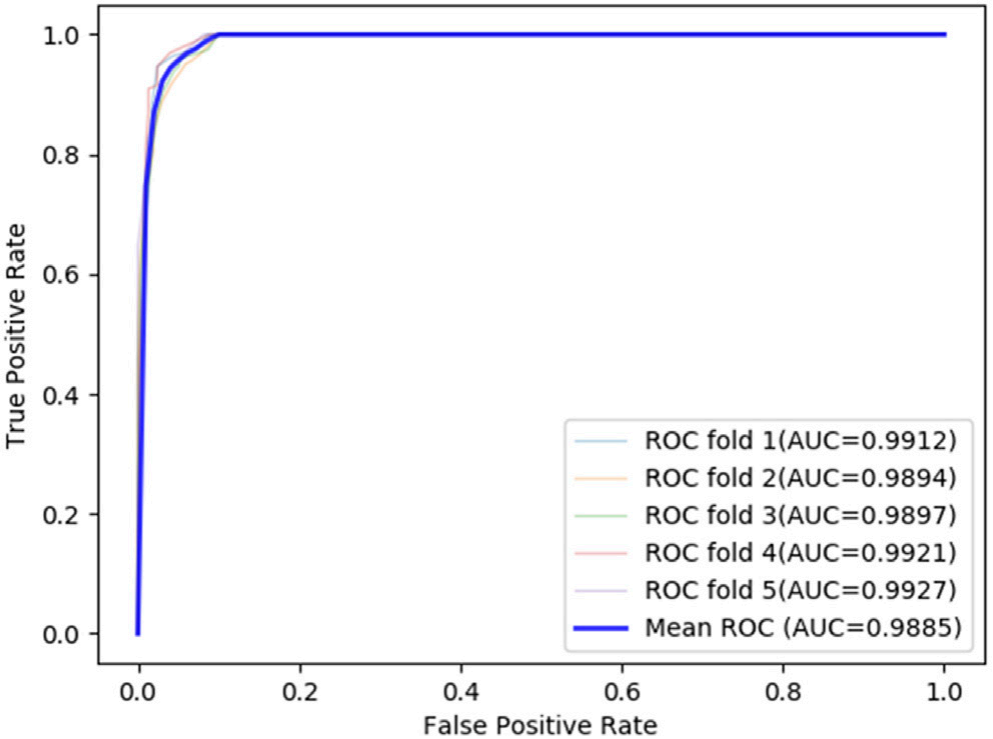
ROC curves of LDA-EAGCN in different situations of 5-fold cross-validation.

To ensure the reliability of the predictive results, a 10-fold cross-validation experiment is employed to evaluate the LDA-EAGCN model, and the total data are divided into 10 parts equally. This 10-fold cross-validation would be cycled 10 times to guarantee each data part is used as a validation set one time. Then a total of 10 training sessions are conducted, and the average model performance is regarded as the final result. The ROC curve is used to evaluate the performance of the LDA-EAGCN model, and it can describe the relationship between the true positive rate (TPR) and false positive rate (FPR) under different thresholds. The larger the area value of AUC under the ROC curve, the better the prediction performance. In the 10-fold cross-validation of the LDA-EAGCN model, the average AUC value reached 0.9854 (Figure 3). We also did a 5-fold cross-validation experiment, and the average AUC value reached 0.9885 (Figure 4).

**FIGURE 5:**
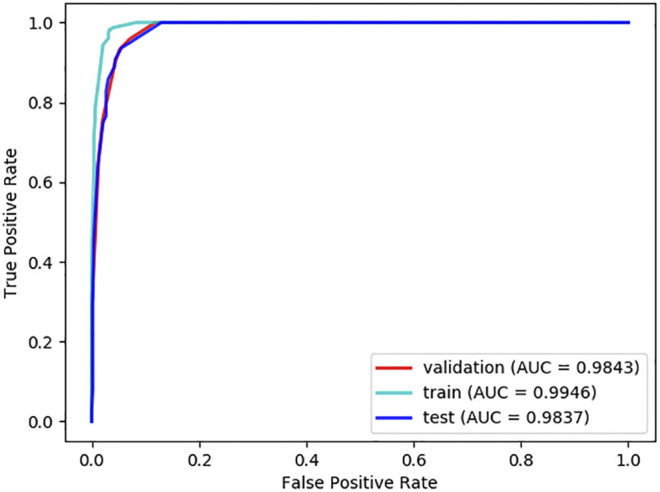
ROC curves of independent testing.

To confirm whether the experimental results of LDA-EAGCN are over fitted, one-tenth of the samples was further separated as an independent dataset, and remaining examples were used for training the classifier in the LDA-EAGCN. The ROC curves of the training set, the testing set, and the validation set are shown in Figure 5. The AUC value of LDA-EAGCN achieved 0.9843 on the validation set, which demonstrated that the excellent performance of 10-fold cross-validations was not generated by overfitting.

In addition, in order to comprehensively evaluate LDA-EAGCN, some metrics, such as accuracy (ACC), sensitivity (SEN), specificity (SPEC), precision (PREC), and Matthews correlation coefficient (MCC), were particularly added. More details of these metrics can be seen in Tables 1–3.

**TABLE 1:** Results of 10-fold cross-validation.

	ACC	SEN	SPEC	PREC	MCC	AUC
1	0.9562	0.9503	0.9618	0.9603	0.9123	0.9911
2	0.9307	1	0.87	0.8707	0.8704	0.9835
3	0.949	0.9489	0.9491	0.9449	0.8979	0.9881
4	0.948	0.9724	0.9255	0.9234	0.8972	0.9886
5	0.9541	0.9706	0.9386	0.9371	0.9088	0.9895
6	0.9582	0.9341	0.9791	0.9748	0.9164	0.9922
7	0.9317	0.934	0.9295	0.9242	0.8633	0.9802
8	0.9602	0.958	0.9624	0.96	0.9204	0.9818
9	0.9317	1	0.8694	0.8748	0.8721	0.9898
10	0.9256	0.9278	0.9234	0.9221	0.8512	0.9811
Mean	0.9445	0.9596	0.9309	0.9292	0.891	0.9853

**TABLE 2:** Results of 5-fold cross-validation.

	ACC	SEN	SPEC	PREC	MCC	AUC
1	0.9734	0.9826	0.9647	0.9638	0.9470	0.994
2	0.9734	0.9762	0.9708	0.9699	0.9469	0.997
3	0.9532	1.0000	0.9072	0.9139	0.9105	0.989
4	0.9447	1.0000	0.8882	0.9015	0.8948	0.987
5	0.9554	0.9594	0.9514	0.9513	0.9108	0.994
Mean	0.9600	0.9897	0.9364	0.9401	0.9220	0.989

**TABLE 3:** Results of independent testing.

	ACC	SEN	SPEC	PREC	MCC	AUC
Validation	0.9352	1.0000	0.8713	0.8829	0.8780	0.9843
Train	0.9734	0.9826	0.9647	0.9638	0.9470	0.9946
Test	0.9307	1.0000	0.8700	0.8707	0.8704	0.9837

In order to prove that each association network has an impact on the performance of the model, each associated network was deleted in turn to build the subgraphs, and the performance of the model was calculated. The results demonstrated that our model achieved the best performance when all associated networks were used for calculation. The detailed results can be seen in Supplementary File S6.

### Comparison With Other Models

In our study, the LDA-EAGCN model was compared with other five state-of-the-art models for lncRNA–disease association prediction including LDA-LNSUBRW (Xie et al., 2020), LDASR (Guo et al., 2019), NCPHLDA (Xie et al., 2019), SDLDA (Zeng et al., 2020), and TPGLDA (Ding et al., 2018). The LDA-LNSUBRW model is an lncRNA–disease association prediction method based on linear neighborhood similarity and unbalanced double random walk; the LDASR model obtains feature vectors by integrating lncRNA Gaussian interaction spectrum kernel similarity, disease semantic similarity, and Gaussian interaction spectrum kernel similarity, and finally uses the rotating forest algorithm for predicting lncRNA–disease associations; NCPHLDA integrates the lncRNA cosine similarity network, disease cosine similarity network, and known lncRNA–disease association network, and predicts by network consensus projection; SDLDA is a hybrid computing framework, which uses singular value decomposition and deep learning to extract linear and non-linear features of lncRNAs and diseases, respectively, and then combines linear and non-linear features training; TPGLDA is a novel lncRNA–disease association prediction method based on lncRNA–disease triad, which combines gene–disease association and lncRNA–disease association. Each model in comparison was trained with the same training set and tested with the same test set in the cross-validation.

**FIGURE 6:**
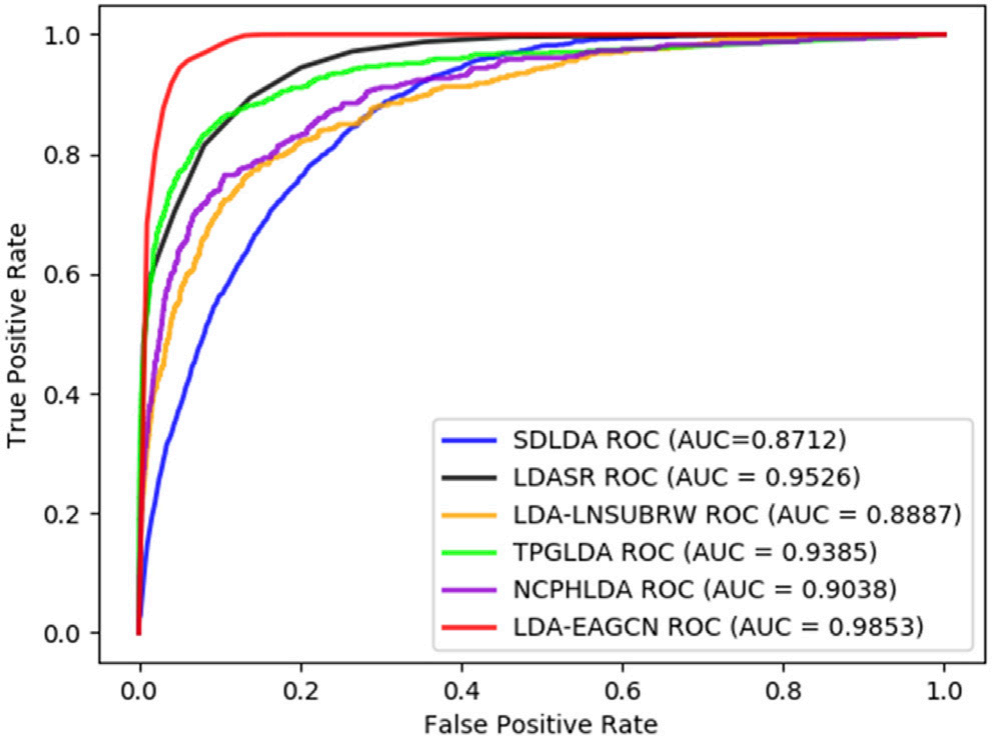
ROC curves of all the models in comparison.

**FIGURE 7:**
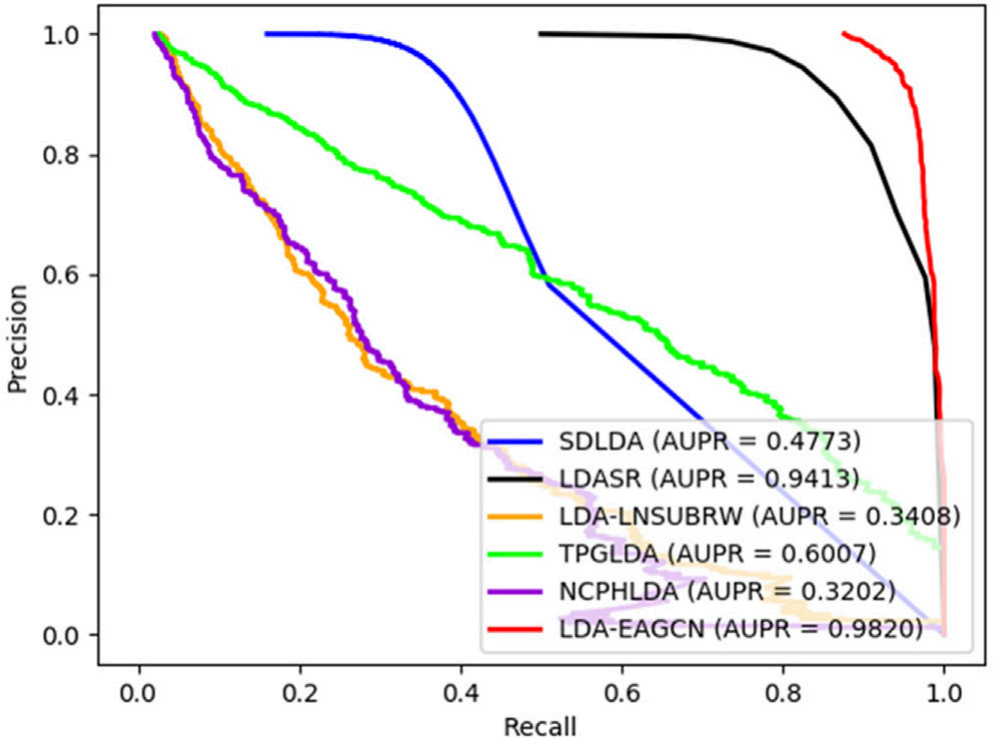
PR curves of all the models in comparison.

The ROC and PR curves of all the models in comparison are given in Figures 6, 7. The AUC values under ROC curve of the LDA-EAGCN model are 0.1141, 0.0317, 0.0966, 0.0468, and 0.0815 higher than those of the SDLDA model, LDASR model, LDA-LNSUBRW model, TPGLDA model, and NCPHLDA model, respectively, which reaches 0.9853. The AUPR values of the LDA-EAGCN model are 0.5047, 0.0407, 0.641, 0.3813, and 0.6618 higher than those of the SDLDA model, LDASR model, LDA-LNSUBRW model, TPGLDA model, and NCPHLDA model, respectively, which reaches 0.9820. The overview of data involved in each comparison model is exhibited in Supplementary File S7.

### Negative Sample Comparison

**FIGURE 8:**
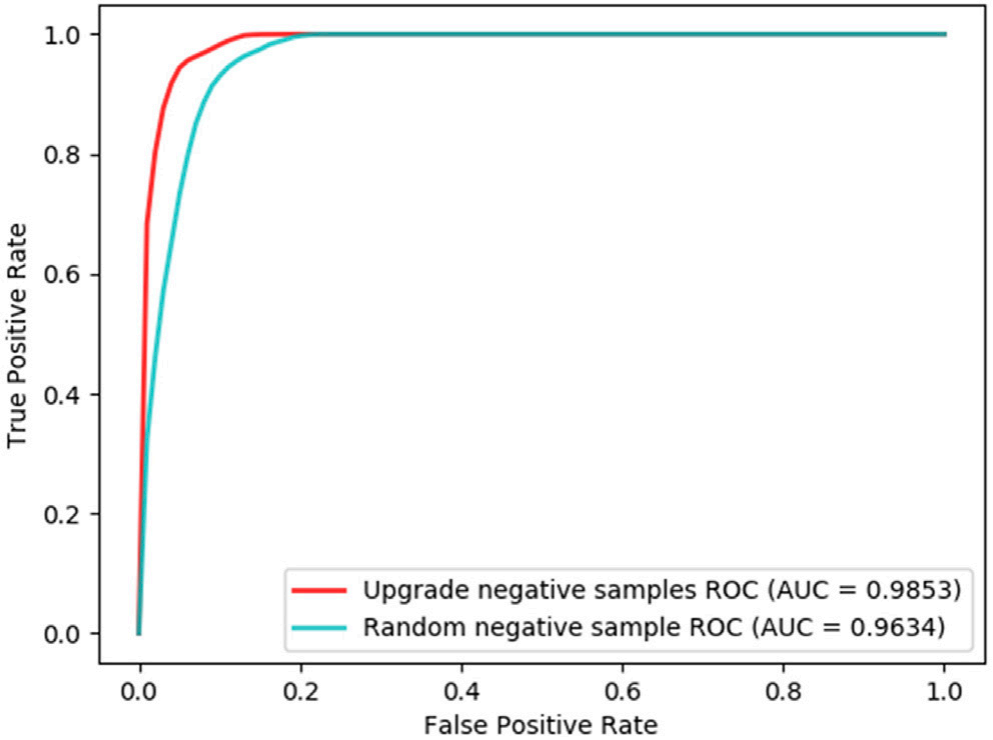
ROC curves of Negative sample comparison.

In order to examine the reliability of the negative samples used in the experiments, the RWRH negative samples, in terms of the associations that have lower scores in the RWRH algorithm, are compared with those randomly selected unknown lncRNA–disease associations. In 10-fold cross-validation, the AUC values of RWRH negative samples and randomly selected negative samples are 0.9853 and 0.9632, respectively (Figure 8). These experiments indicate the reliability of the method for generating negative samples in LDA-EAGCN.

### Case Studies

In order to further demonstrate the predictive ability of the LDA-EAGCN model, case studies were performed over kidney cancer, laryngeal cancer, and liver cancer. First, 4715 pairs of known lncRNA–disease associations and the equivalent generated negative samples were adopted for model training. Then the weight graph of the closest nodes in the spatial contextual of these three diseases and lncRNAs with the unknown associations related with the three diseases are generated, respectively, which are used as the input of LDA-EAGCN. The predictive correlation scores of unknown lncRNA–disease associations between the interested diseases and their unknown lncRNAs are gained. Finally, the predictive correlation scores are sorted in a descending order, and the top 10 lncRNAs with the highest scores of these three diseases are document mined. Among the top ten lncRNAs corresponding to renal cell carcinoma, laryngeal cancer, and liver cancer, eight lncRNAs associated with each disease are supported by recent biological experiments’ literature works, which indicate the LDA-EAGCN model has good performance in predicting unknown relationships. The scores of each lncRNA–disease pair in the experimental data are available in Supplementary File S8.

Kidney neoplasm is a cancer that originates from kidney tissues, which is one of the ten most common cancers, and renal cell carcinoma composes the vast majority of kidney cancer cases (Linehan and Rathmell, 2012). Despite expending high efforts to study kidney neoplasms in biogenetics, there are still great doubts about the occurrence of kidney neoplasms. In order to confirm the validity of the model, LDA-EAGCN was implemented to predict potential kidney neoplasm–related lncRNAs. As a result, eight out of top ten potential lncRNAs related with kidney neoplasms have been validated by recent biological experiments’ literature works (Table 4), which were ranked 1st, 2nd, 3rd, 4th, 6th, 7th, 9th, and 10th in the prediction results, respectively. For example, recent studies have found that CDKN2B-AS1 can be used as a biomarker for poor prognosis of kidney neoplasms (Angenard et al., 2019), DUXAP8 enhances the progression of kidney neoplasms by downregulating miR-126 (Huang et al., 2018), and HOTAIRM1 is downregulated in kidney neoplasms and inhibits hypoxia (Hamilton et al., 2020).

**TABLE 4:** Case study results of kidney neoplasms.

Rank	LncRNA	Evidence (PMID)
1	CDKN2B-AS1	31040073
2	DUXAP8	30317248
3	HOTAIRM1	31862408
4	MIAT	30041179
5	SNHG16	Without evidence
6	NEAT1	30622287
7	SNHG12	32641718
8	SNHG5	Without evidence
9	SOX9	28118628
10	TUG1	30132963

Laryngeal neoplasm is a common malignant tumor that accounts for 4.5% of systemic malignancies, and it is also the second largest malignant tumor of head and neck malignant tumors (Obid et al., 2019). The loss of laryngeal function will greatly affect language expression and swallowing function with some special senses. Therefore, it is imperative to identify novel lncRNAs for early diagnosis, prognosis, and treatment of laryngeal neoplasms. Accumulating evidence has demonstrated that lncRNAs have played critical roles in the development and progression of laryngeal neoplasms (Xiang et al., 2019; Zhang G et al., 2019; Li et al., 2020). LDA-EAGCN was further implemented to identify lncRNAs associated with laryngeal neoplasms. As a result, eight out of top ten potential lncRNAs related with laryngeal neoplasms have also been validated by recent biological experiments’ literature works (Table 5), which were ranked 1st, 2nd, 3rd, 4th, 5th, 7th, 8th, and 9th in the prediction results, respectively. For example, CDKN2B-AS1 regulates the cell cycle of laryngeal neoplasms (F. Liu et al., 2020), PVT1 regulates miR-519d-3p to promote the development of laryngeal neoplasms (Zheng et al., 2019), and CCAT1 regulates the progression of laryngeal neoplasms (Zhang and Hu, 2017) through different ways. Notably, the model predicts that lncRNA GAS5, which scored second, inhibits proliferation and metastasis of laryngeal neoplasms by regulating the PI3K/AKT/mTOR signaling pathway, according to a recent study in 2020 (Liu et al., 2021).

**TABLE 5:** Case study results of laryngeal cancer.

Rank	LncRNA	Evidence (PMID)
1	CDKN2B-AS1	31960744
2	GAS5	33641529
3	PVT1	30304557
4	CCAT1	28631575
5	H19	26872375
6	HULC	Without evidence
7	MALAT1	31792655, 31837057
8	MEG3	31328388, 30915750
9	NEAT1	26822763, 31364125
10	SNHG7	Without evidence

Liver neoplasm is a common malignant cancer globally, and it is the second leading cause of cancer death worldwide (Yamashita and Kaneko, 2016). Liver neoplasms are a special kind of cancer, and their occurrence and development rate often depend on the host, disease, and environmental factors and their complex interactions. Numerous experimental results prove that the development and progression of liver neoplasms are closely related to the mutations and dysregulations of some lncRNAs (Wang et al., 2017; Zhang Z et al., 2019; Zhang et al., 2020). LDA-EAGCN is applied to liver neoplasms for potentially related lncRNA prediction. By mining recent biological experiments’ literature works, eight out of top ten potential lncRNAs related with liver neoplasms are validated (Table 6), which were ranked 1st, 2nd, 3rd, 5th, 6th, 8th, 9th, and 10th in the prediction results, respectively. For example, BANCR can be used as a potential therapeutic target for liver neoplasms (Zhou and Gao, 2016), NEAT1 is necessary for liver neoplasm marker CD44 expression (Koyama et al., 2020), and LINC00473 promotes the progression of liver cancer by acting as microRNA-195 ceRNA and increasing HMGA2 expression (Mo et al., 2019).

**TABLE 6:** Case study results of liver cancer.

Rank	LncRNA	Evidence (PMID)
1	BANCR	26758762
2	NEAT1	32168951
3	CYTOR	32918630
4	MIR100HG	Without evidence
5	AFAP1-AS1	29057544
6	CCAT1	30773676
7	LINC-PINT	Without evidence
8	CASC2	31301261
9	HOXA-AS2	27855366
10	LINC00473	31562977

## Discussion

In this study, a model based on close node weight graph of the spatial neighborhood and edge attention graph convolutional networks was proposed to predict disease-related lncRNAs by multisource data. Inspired by the great success of the EAGCN method on the chemical molecule property recognition problem, the prediction of lncRNA–disease associations could be regarded as a component recognition problem of the lncRNA–disease characteristic graph. The CNWGSN features of lncRNA–disease associations combined with known lncRNA–disease associations have been introduced to train the EAGCN method, and the correlation scores of input data were predicted with EAGCN for judging whether the input lncRNAs are associated with the input diseases.

In order to excavate core features of lncRNA–diseases relationship in a graph and remove redundancy, the closest node weight graph of the spatial neighborhoods (CNWGSNs) of lncRNA–disease associations was constructed. It not only considers the features of disease–disease relationship, lncRNA–lncRNA relationship, and the association between disease and lncRNA but also considers the features of lncRNA and disease in a multidimensional space. In addition, CNWGSN can also provide a great logic and mathematical support for EAGCN to learn and summarize the internal relationship between lncRNA and disease. Then the features of lncRNA–disease are trained into the edge attention-based multi-relational graph convolutional networks (EAGCNs), which accurately learn multiple edge relations in multiple graphs. For solving the problem of missing negative samples, the RWRH algorithm is adopted to randomly select lncRNA–disease pairs with low correlation scores as negative samples.

Our model LDA-EAGCN gets better performance in the 10-fold cross-over test, and the mean AUC of it reached 0.9853, which is higher than that of other five state-of-the-art models. As for the experiments of case studies, in the top ten lncRNAs of kidney cancer, laryngeal cancer, and liver cancer, 24 of all 30 lncRNAs were verified to be associated with the diseases.

Although the model can achieve good results, there is still room for improvement. At present, the model only uses lncRNA–disease data, and more types of biological data and more elaborately designed fusion methods can be applied in the future.

## Data Availability

The original contributions presented in the study are included in the article/Supplementary Material, further inquiries can be directed to the corresponding authors.

## Supplementary Material

Supplementary File 1 Summarization of lncRNA–disease prediction models.

Supplementary File 2 Disease semantic similarity scores.

Supplementary File 3 LncRNA functional similarity scores.

Supplementary File 4 LncRNA–disease correlation scores of RWRH.

Supplementary File 5 Experimental details.

Supplementary File 6 Details of deleted associated networks.

Supplementary File 7 Details of data involved in each comparison model.

Supplementary File 8 LncRNA–disease correlation scores of LDA-EAGCN predictions.

## References

[B1] Angenard G. Merdrignac A. Louis C. Edeline J. Coulouarn C. (2019). Expression of Long Non-coding RNA ANRIL Predicts a Poor Prognosis in Intrahepatic Cholangiocarcinoma. Dig. Liver Dis. 51 (9), 1337–1343. 10.1016/j.dld.2019.03.019 31040073

[B2] Chen X. Yan G.-Y. (2013). Novel Human lncRNA-Disease Association Inference Based on lncRNA Expression Profiles. Bioinformatics 29 (20), 2617–2624. 10.1093/bioinformatics/btt426 24002109

[B3] Chen G. Wang Z. Wang D. Qiu C. Liu M. Chen X. (2013). LncRNADisease: a Database for Long-Non-Coding RNA-Associated Diseases. Nucleic Acids Res. 41 (Database issue), D983–D986. 10.1093/nar/gks1099 23175614 PMC3531173

[B4] Chen X. Clarence Yan C. Luo C. Ji W. Zhang Y. Dai Q. (2015). Constructing lncRNA Functional Similarity Network Based on lncRNA-Disease Associations and Disease Semantic Similarity. Sci. Rep. 5, 11338. 10.1038/srep11338 26061969 PMC4462156

[B5] Chen X. You Z.-H. Yan G.-Y. Gong D.-W. (2016). IRWRLDA: Improved Random Walk with Restart for lncRNA-Disease Association Prediction. Oncotarget 7 (36), 57919–57931. 10.18632/oncotarget.11141 27517318 PMC5295400

[B6] Ding L. Wang M. Sun D. Li A. (2018). TPGLDA: Novel Prediction of Associations between lncRNAs and Diseases via lncRNA-Disease-Gene Tripartite Graph. Sci. Rep. 8 (1), 1065. 10.1038/s41598-018-19357-3 29348552 PMC5773503

[B7] Fu G. Wang J. Domeniconi C. Yu G. (2018). Matrix Factorization-Based Data Fusion for the Prediction of lncRNA-Disease Associations. Bioinformatics 34 (9), 1529–1537. 10.1093/bioinformatics/btx794 29228285

[B8] Guo Z.-H. You Z.-H. Wang Y.-B. Yi H.-C. Chen Z.-H. (2019). A Learning-Based Method for LncRNA-Disease Association Identification Combing Similarity Information and Rotation Forest. iScience 19, 786–795. 10.1016/j.isci.2019.08.030 31494494 PMC6733997

[B9] Guttman M. Russell P. Ingolia N. T. Weissman J. S. Lander E. S. (2013). Ribosome Profiling Provides Evidence that Large Noncoding RNAs Do Not Encode Proteins. Cell 154 (1), 240–251. 10.1016/j.cell.2013.06.009 23810193 PMC3756563

[B10] Hamilton M. J. Young M. Jang K. Sauer S. Neang V. E. King A. T. (2020). HOTAIRM1 lncRNA Is Downregulated in clear Cell Renal Cell Carcinoma and Inhibits the Hypoxia Pathway. Cancer Lett. 472, 50–58. 10.1016/j.canlet.2019.12.022 31862408 PMC6992348

[B11] Huang T. Wang X. Yang X. Ji J. Wang Q. Yue X. (2018). Long Non-coding RNA DUXAP8 Enhances Renal Cell Carcinoma Progression via Downregulating miR-126. Med. Sci. Monit. 24, 7340–7347. 10.12659/msm.910054 30317248 PMC6198709

[B12] Kapranov P. Cheng J. Dike S. Nix D. A. Duttagupta R. Willingham A. T. (2007). RNA Maps Reveal New RNA Classes and a Possible Function for Pervasive Transcription. Science 316 (5830), 1484–1488. 10.1126/science.1138341 17510325

[B13] Koyama S. Tsuchiya H. Amisaki M. Sakaguchi H. Honjo S. Fujiwara Y. (2020). NEAT1 Is Required for the Expression of the Liver Cancer Stem Cell Marker CD44. Int. J. Mol. Sci. 21 (6), 1927. 10.3390/ijms21061927 PMC7139689 32168951

[B14] Lan W. Li M. Zhao K. Liu J. Wu F.-X. Pan Y. (2017). LDAP: a Web Server for lncRNA-Disease Association Prediction. Bioinformatics 33 (3), btw639–460. 10.1093/bioinformatics/btw639 28172495

[B49] Li Y. Patra J. C. (2010). Genome-Wide Inferring Gene-Henotype Relationship by Walking on the Heterogeneous Network. Bioinformatics 26 (9), 1219–1224. 10.1093/bioinformatics/btq108 20215462

[B15] Li G. Pan C. Sun J. Wan G. Sun J. (2020). lncRNA SOX2OT Regulates Laryngeal Cancer Cell Proliferation, Migration and Invasion and Induces Apoptosis by Suppressing miR654. Exp. Ther. Med. 19 (5), 3316–3324. 10.3892/etm.2020.8577 32266028 PMC7132247

[B16] Liao Q. Liu C. Yuan X. Kang S. Miao R. Xiao H. (2011). Large-scale Prediction of Long Non-coding RNA Functions in a Coding-Non-Coding Gene Co-expression Network. Nucleic Acids Res. 39 (9), 3864–3878. 10.1093/nar/gkq1348 21247874 PMC3089475

[B17] Linehan W. M. Rathmell W. K. (2012). Kidney Cancer. Urol. Oncol. Semin. Original Invest. 30 (6), 948–951. 10.1016/j.urolonc.2012.08.021 PMC4419144 23218074

[B18] Liu F. Xiao Y. Ma L. Wang J. (2020). Regulating of Cell Cycle Progression by the lncRNA CDKN2B-AS1/miR-324-5p/ROCK1 axis in Laryngeal Squamous Cell Cancer. Int. J. Biol. Markers 35 (1), 47–56. 10.1177/1724600819898489 31960744

[B19] Liu W. Zhan J. Zhong R. Li R. Sheng X. Xu M. (2021). Upregulation of Long Noncoding RNA_GAS5 Suppresses Cell Proliferation and Metastasis in Laryngeal Cancer via Regulating PI3K/AKT/mTOR Signaling Pathway. Technol. Cancer Res. Treat. 20, 153303382199007. 10.1177/1533033821990074 PMC7923983 33641529

[B20] Lu C. Yang M. Luo F. Wu F.-X. Li M. Pan Y. (2018). Prediction of lncRNA-Disease Associations Based on Inductive Matrix Completion. Bioinformatics 34 (19), 3357–3364. 10.1093/bioinformatics/bty327 29718113

[B21] Mercer T. R. Dinger M. E. Mattick J. S. (2009). Long Non-coding RNAs: Insights into Functions. Nat. Rev. Genet. 10 (3), 155–159. 10.1038/nrg2521 19188922

[B22] Mo J. Li B. Zhou Y. Xu Y. Jiang H. Cheng X. (2019). LINC00473 Promotes Hepatocellular Carcinoma Progression via Acting as a ceRNA for microRNA-195 and Increasing HMGA2 Expression. Biomed. Pharmacother. 120, 109403. 10.1016/j.biopha.2019.109403 31562977

[B23] Nelson S. J. Johnston W. D. Humphreys B. L. (2001). “Relationships in Medical Subject Headings (MeSH): Relationships in the Organization of Knowledge,” in Relationships in the Organization of Knowledge. New York, NY: Kluwer Academic Publishers, 171–184. 10.1007/978-94-015-9696-1_11

[B24] Ning S. Zhang J. Wang P. Zhi H. Wang J. Liu Y. (2016). Lnc2Cancer: a Manually Curated Database of Experimentally Supported lncRNAs Associated with Various Human Cancers. Nucleic Acids Res. 44 (D1), D980–D985. 10.1093/nar/gkv1094 26481356 PMC4702799

[B25] Obid R. Redlich M. Tomeh C. (2019). The Treatment of Laryngeal Cancer. Oral. Maxillofac. Surg. Clin. North Am. 31 (1), 1–11. 10.1016/j.coms.2018.09.001 30449522

[B26] Ponting C. P. Oliver P. L. Reik W. (2009). Evolution and Functions of Long Noncoding RNAs. Cell 136 (4), 629–641. 10.1016/j.cell.2009.02.006 19239885

[B27] Shang C. Liu Q. Chen K. S. Sun J. Lu J. Yi J. (2018). Edge Attention-Based Multi-Relational Graph Convolutional Networks. ArXiv, abs/1802.04944.

[B28] Sheng N. Cui H. Zhang T. Xuan P. (2021). Attentional Multi-Level Representation Encoding Based on Convolutional and Variance Autoencoders for lncRNA-Disease Association Prediction. Brief Bioinform. 22 (3), 1–14. 10.1093/bib/bbaa067 32444875

[B29] Sun J. Shi H. Wang Z. Zhang C. Liu L. Wang L. (2014). Inferring Novel lncRNA-Disease Associations Based on a Random Walk Model of a lncRNA Functional Similarity Network. Mol. Biosyst. 10 (8), 2074–2081. 10.1039/c3mb70608g 24850297

[B30] Wang K. C. Chang H. Y. (2011). Molecular Mechanisms of Long Noncoding RNAs. Mol. Cel 43 (6), 904–914. 10.1016/j.molcel.2011.08.018 PMC3199020 21925379

[B31] Wang D. Wang J. Lu M. Song F. Cui Q. (2010). Inferring the Human microRNA Functional Similarity and Functional Network Based on microRNA-Associated Diseases. Bioinformatics 26 (13), 1644–1650. 10.1093/bioinformatics/btq241 20439255

[B32] Wang H. Huo X. Yang X.-R. He J. Cheng L. Wang N. (2017). STAT3-mediated Upregulation of lncRNA HOXD-AS1 as a ceRNA Facilitates Liver Cancer Metastasis by Regulating SOX4. Mol. Cancer 16 (1), 136. 10.1186/s12943-017-0680-1 28810927 PMC5558651

[B33] Wang L. Xiao Y. Li J. Feng X. Li Q. Yang J. (2019). IIRWR: Internal Inclined Random Walk with Restart for LncRNA-Disease Association Prediction. IEEE Access 7, 54034–54041. 10.1109/ACCESS.2019.2912945

[B34] Wu X. Lan W. Chen Q. Dong Y. Liu J. Peng W. (2020). Inferring LncRNA-Disease Associations Based on Graph Autoencoder Matrix Completion. Comput. Biol. Chem. 87, 107282. 10.1016/j.compbiolchem.2020.107282 32502934

[B35] Xiang Y. Li C. Liao Y. Wu J. (2019). An Integrated mRNA‐lncRNA Signature for Relapse Prediction in Laryngeal Cancer. J. Cel Biochem. 120 (9), 15883–15890. 10.1002/jcb.28859 31062433

[B36] Xie G. Huang Z. Liu Z. Lin Z. Ma L. (2019). NCPHLDA: a Novel Method for Human lncRNA-Disease Association Prediction Based on Network Consistency Projection. Mol. Omics 15 (6), 442–450. 10.1039/c9mo00092e 31686064

[B37] Xie G. Jiang J. Sun Y. (2020). LDA-LNSUBRW: lncRNA-Disease Association Prediction Based on Linear Neighborhood Similarity and Unbalanced Bi-random Walk. IEEE/ACM Trans. Comput. Biol. Bioinf. PP, 1–1. 10.1109/tcbb.2020.3020595 32870798

[B38] Xuan P. Cao Y. Zhang T. Kong R. Zhang Z. (2019). Dual Convolutional Neural Networks with Attention Mechanisms Based Method for Predicting Disease-Related lncRNA Genes. Front. Genet. 10, 416. 10.3389/fgene.2019.00416 31130990 PMC6509943

[B39] Yamashita T. Kaneko S. (2016). Liver Cancer. Rinsho Byori 64 (7), 787–796. 30695467

[B40] Yang X. Gao L. Guo X. Shi X. Wu H. Song F. (2014). A Network Based Method for Analysis of lncRNA-Disease Associations and Prediction of lncRNAs Implicated in Diseases. PLoS One 9 (1), e87797. 10.1371/journal.pone.0087797 24498199 PMC3909255

[B41] Zeng M. Lu C. Zhang F. Li Y. Wu F.-X. Li Y. (2020). SDLDA: lncRNA-Disease Association Prediction Based on Singular Value Decomposition and Deep Learning. Methods 179, 73–80. 10.1016/j.ymeth.2020.05.002 32387314

[B42] Zhang Y. Hu H. (2017). Long Non-coding RNA CCAT1/miR-218/ZFX axis Modulates the Progression of Laryngeal Squamous Cell Cancer. Tumour Biol. 39 (6), 101042831769941. 10.1177/1010428317699417 28631575

[B43] Zhang Z. Wang S. Yang F. Meng Z. Liu Y. (2020). LncRNA ROR1AS1 High Expression and its Prognostic Significance in Liver Cancer. Oncol. Rep. 43 (1), 55–74. 10.3892/or.2019.7398 31746401 PMC6908930

[B44] Zhang G G. Fan E. Zhong Q. Feng G. Shuai Y. Wu M. (2019). Identification and Potential Mechanisms of a 4-lncRNA Signature that Predicts Prognosis in Patients with Laryngeal Cancer. Hum. Genomics 13 (1), 36. 10.1186/s40246-019-0230-6 31416476 PMC6694645

[B45] Zhang Z Z. Wang S. Liu Y. Meng Z. Chen F. (2019). Low lncRNA ZNF385D-AS2 E-xpression and its P-rognostic S-ignificance in L-iver C-ancer. Oncol. Rep. 42 (3), 1110–1124. 10.3892/or.2019.7238 31322274 PMC6667919

[B46] Zhao T. Xu J. Liu L. Bai J. Xu C. Xiao Y. (2015). Identification of Cancer-Related lncRNAs through Integrating Genome, Regulome and Transcriptome Features. Mol. Biosyst. 11 (1), 126–136. 10.1039/c4mb00478g 25354589

[B47] Zheng X. Zhao K. Liu T. Liu L. Zhou C. Xu M. (2019). Long Noncoding RNA PVT1 Promotes Laryngeal Squamous Cell Carcinoma Development by Acting as a Molecular Sponge to Regulate miR‐519d‐3p. J. Cel Biochem. 120 (3), 3911–3921. 10.1002/jcb.27673 30304557

[B48] Zhou T. Gao Y. (2016). Increased Expression of LncRNA BANCR and its Prognostic Significance in Human Hepatocellular Carcinoma. World J. Surg. Onc 14 (1), 8. 10.1186/s12957-015-0757-5 PMC4709863 26758762

