# Supplementary material for: Prediction of lncRNA–Disease Associations via Closest Node Weight Graphs of the Spatial Neighborhood Based on the Edge Attention Graph Convolutional Network
Source: Front Genet. 2022 Jan 4;12:808962. doi: 10.3389/fgene.2021.808962 (PMC8763691; doi:10.3389/fgene.2021.808962)
Supplement: Supplementary file 3 [file Table1.DOCX]

**The Summarization of LncRNA-Disease Prediction Models**

| Categories | PMID / DOI | Model name | Introduction |
| --- | --- | --- | --- |
| Based biological networks | 21247874 | CNC co-expression network | A coding-non-coding gene co-expression network constructed for predicting probable functions. |
|  | 24498199 | CNC gene-disease bipartite network | A network based method for analysis of lncRNA-disease associations and prediction of lncRNAs implicated in diseases. |
|  | 24850297 | RWRlncD | Inferring novel lncRNA-disease associations based on a random walk model of a lncRNA functional similarity network. |
|  | 27517318 | IRWRLDA | Improved random walk with restart for lncRNA-disease association prediction. |
|  | 29228285 | MFLDA | Matrix factorization-based data fusion for the prediction of lncRNA-disease associations. |
|  | 29348552 | TPGLDA | Novel prediction of associations between lncRNAs and diseases via lncRNA-disease-gene tripartite graph. |
|  | DOI:10.1109/ACCESS.2019.2912945 | IIRWR | Internal Inclined Random Walk With Restart for LncRNA-Disease Association Prediction. |
|  | 31686064 | NCPHLDA | A novel method for human lncRNA-disease association prediction based on network consistency projection. |
|  | 32870798 | LDA-LNSUBRW | LncRNA-disease association prediction based on linear neighborhood similarity and unbalanced bi-random walk. |
| Based on Machine Learning or Deep Learning methods | 24002109 | LRLSLDA | Novel human lncRNA-disease association inference based on lncRNA expression profiles. |
|  | 25354589 |  | Identification of cancer-related lncRNAs through integrating genome, regulome and transcriptome features. |
|  | 26061969 | LNCSIM | Constructing lncRNA functional similarity network based on lncRNA-disease associations and disease semantic similarity. |
|  | DOI:10.1109/BIBM47256.2019.8983279 | TVWS | Combined matrix factorization method with a two- hidden-layer neural network model to predicted lncRNA-disease associations. |
|  | 28172495 | LDAP | A web server for lncRNA-disease association prediction.. |
|  | 29718113 | SIMCLDA | Prediction of lncRNA-disease associations based on inductive matrix completion.. |
|  | 31494494 | LDASR | A learning-based method for lncRNA-disease association identification combing similarity information and Rotation Forest. |
|  | 31130990 | CNNLDA | Dual convolutional neural networks with attention mechanisms based method for predicting disease-related lncRNA genes. |
|  | 32387314 | SDLDA | lncRNA-disease association prediction based on singular value decomposition and deep learning. |
|  | 32444875 | VADLP | Attentional multi-level representation encoding based on convolutional and variance autoencoders for lncRNA-disease association prediction. |
|  | 32502934 | GAMCLDA | Inferring LncRNA-disease associations based on graph autoencoder matrix completion. |
