# Supplementary material for: Prediction of lncRNA–Disease Associations via Closest Node Weight Graphs of the Spatial Neighborhood Based on the Edge Attention Graph Convolutional Network
Source: Front Genet. 2022 Jan 4;12:808962. doi: 10.3389/fgene.2021.808962 (PMC8763691; doi:10.3389/fgene.2021.808962)
Supplement: Supplementary file 5 [file Table4.DOCX]

**Experimental code details of LDA-EAGCN**

**Part 1. Details of cross-validation experiments:**

Number of positive samples: 4518

Number of negative samples: 4518

1. 10-fold cross validation

Train Set Samples : Validation Set Samples = 8132 : 904

2. 5-fold cross validation

Train Set Samples : Validation Set Samples = 7229 : 1807

All classifiers' parameters were set as defaults in Edge Attention-based multi-Relational Graph Convolutional Networks.

**Part 2. Table S1. Python and packages versions for LDA-EAGCN model**

| **Python and Package Name** | **Python and Package Version** |
| --- | --- |
| Python | 3.6.x |
| networkx | 2.5.1 |
| numpy | 1.19.5 |
| pandas | 0.20.3 |
| scikit-learn | 0.24.2 |
| scipy | 1.5.4 |
| xgboost | 1.3.3 |
